# Immunological characterization of a VIR protein family member (VIR-14) in *Plasmodium vivax*-infected subjects from different epidemiological regions in Africa and South America

**DOI:** 10.1371/journal.pntd.0011229

**Published:** 2023-04-07

**Authors:** Raianna F. Fantin, Camila H. Coelho, Anne D. Berhe, Luisa M. D. Magalhães, Dhélio B. Pereira, Nichole D. Salinas, Niraj H. Tolia, Chanaki Amaratunga, Seila Suon, Issaka Sagara, David L. Narum, Ricardo T. Fujiwara, Claudia Abejon, Antonio Campos-Neto, Patrick E. Duffy, Lilian L. Bueno

**Affiliations:** 1 Laboratory of Malaria Immunology and Vaccinology, National Institute of Allergy and Infectious Diseases, National Institutes of Health (NIH), Bethesda, Maryland, United States of America; 2 School of Medicine, Federal University of Minas Gerais, Belo Horizonte, Brazil; 3 Department of Parasitology, Federal University of Minas Gerais, Belo Horizonte, Brazil; 4 Center for Research in Tropical Medicine, Porto Velho, Brazil; 5 National Center for Parasitology, Entomology, and Malaria Control, Phnom Penh, Cambodia; 6 Faculty of Medicine and Odonto-Stomatology, University of Sciences, Techniques and Technology of Bamako, Bamako, Mali; 7 DetectoGen Inc., Westborough, Massachusetts, United States of America; 8 Cummings School of Veterinary Medicine, Tufts University, North Grafton, Massachusetts, United States of America; Ohio State University, UNITED STATES

## Abstract

*Plasmodium vivax* is a major challenge for malaria control due to its wide geographic distribution, high frequency of submicroscopic infections, and ability to induce relapses due to the latent forms present in the liver (hypnozoites). Deepening our knowledge of parasite biology and its molecular components is key to develop new tools for malaria control and elimination. This study aims to investigate and characterize a *P*. *vivax* protein (*Pv*Vir14) for its role in parasite biology and its interactions with the immune system. We collected sera or plasma from *P*.*vivax*-infected subjects in Brazil (n = 121) and Cambodia (n = 55), and from *P*. *falciparum-*infected subjects in Mali (n = 28), to assess antibody recognition of *Pv*Vir14. Circulating antibodies against *Pv*Vir14 appeared in 61% and 34.5% of subjects from Brazil and Cambodia, respectively, versus none (0%) of the *P*. *falciparum*-infected subjects from Mali who have no exposure to *P*. *vivax*. IgG1 and IgG3 most frequently contributed to anti-*Pv*Vir14 responses. *Pv*Vir14 antibody levels correlated with those against other well-characterized sporozoite/liver (*Pv*CSP) and blood stage (*Pv*DBP-RII) antigens, which were recognized by 7.6% and 42% of Brazilians, respectively. Concerning the cellular immune profiling of Brazilian subjects, *Pv*Vir14 seroreactive individuals displayed significantly higher levels of circulating atypical (CD21^−^ CD27^−^) B cells, raising the possibility that atypical B cells may be contribute to the *Pv*Vir14 antibody response. When analyzed at a single-cell level, the B cell receptor gene hIGHV3-23 was only seen in subjects with active *P*.*vivax* infection where it comprised 20% of V gene usage. Among T cells, CD4^+^ and CD8^+^ levels differed (lower and higher, respectively) between subjects with versus without antibodies to *Pv*Vir14, while NKT cell levels were higher in those without antibodies. Specific B cell subsets, anti-*Pv*Vir14 circulating antibodies, and NKT cell levels declined after treatment of *P*. *vivax*. This study provides the immunological characterization of *Pv*Vir14, a unique *P*. *vivax* protein, and possible association with acute host’s immune responses, providing new information of specific host-parasite interaction.

**Trial registration**: TrialClinicalTrials.gov Identifier: NCT00663546 & ClinicalTrials.gov NCT02334462.

## Author summary

*Plasmodium vivax* is one of the main species causing malaria around the globe, thus the development of control tools are needed if progress is to be made towards malaria elimination. Among them, vaccines are a crucial one, since it’s been proven that a well-established immunization program can save millions of lives a year. Although the development of vaccines against malaria have had substantial progress in the last decade, most of the efforts are concentrated towards *Plasmodium falciparum*. In this study, the authors aimed to investigate the potential role of a recently *P*. *vivax*-protein (*Pv*Vir14) discovered in the urine of infected subjects from the Brazilian Amazon. Initial findings showed that, although the protein elicits a higher frequency of antibodies among the Brazilian population (where it was first discovered), antibody titers were also found within African populations exposed and/or infected by *P*. *Vivax*. Interestingly, populations exposed to *P*. *falciparum* only did not have any immune responses against the protein, indicating that it is exclusive to vivax. Discovering key antigens is one of the most important steps towards the development of control tools. These findings provide the first characterization of naturally acquired antibody responses from different malaria regions against a new and promising target.

## Introduction

Despite numerous efforts to eradicate malaria, this disease is still a major cause of mortality and morbidity worldwide that is endemic in Sub-Saharan Africa, Southeast Asia, Middle East, Oceania, and Latin America. In 2021, an estimated 247 million cases of malaria occurred worldwide [1]. Specifically in Brazil, around 140,000 cases were registered in 2021 [2], the vast majority in the Amazon region. *Plasmodium vivax* is the most common human malaria species in Brazil, causing approximately 85% of the cases [2]. For many years, *P*. *vivax* infection was considered to be benign and often asymptomatic, but in recent years, severe cases of *P*. *vivax* malaria have been reported more frequently, not only in Brazil but in several endemic regions worldwide [3–5]. Among the symptoms associated with severe vivax malaria, the most common are anemia, thrombocytopenia, renal and hepatic dysfunction [6].

The recent increase in drug-resistant *P*. *vivax* strains [7], the evolution toward more virulent forms [8], the early production of gametocytes as well as the formation of hypnozoites with relapse potential [9], make *P*. *vivax* malaria a public health issue of increasing importance. An efficacious vaccine targeting *P*. *vivax* would be a potent and cost-effective tool to reduce transmission, and thus an important measure to control malaria [10]. However, despite its wide distribution and new awareness of its virulence, minimal investments have been made in vaccine discovery for *P*. *vivax* compared with those for *P*. *falciparum*.

An efficient vaccine will protect from *P*. *vivax* through targeting sites of vulnerability or mechanisms of parasite virulence [11]. The Vir superfamily of *P*. *vivax* encompasses numerous surface proteins that have been related to parasite virulence [12,13], and associated with escape mechanisms through antigenic variation [14]. Significantly, proteins belonging to the Vir superfamily are exported to the surface of infected reticulocytes and can play a role in partial adhesion to host endothelial receptors, making these proteins accessible for immune recognition as well [15]. In prior studies, antibodies against Vir have been characterized as markers of exposure at a population level with a tendency to increase during acute infections [16]. Recently, a unique protein from *P*. *vivax*, named *Pv*Vir14 (from the Vir superfamily), was identified circulating in high levels during acute infection and recognized by *P*. *vivax* malaria patients [17].

Here, we determined that specific antibodies targeting the *Pv*Vir14 protein were present in a high proportion of sera/plasma from *P*. *vivax*-infected subjects in Brazil and in Cambodia. Considering the absence of previous immunological studies comparing different epidemiological regions, we characterized immune responses to *Pv*Vir14 at a serum and cellular levels to identify signatures of immunogenicity and assess the potential value of *Pv*Vir14 as a protein of interest.

## Methods

### Ethics statement

The studies involving human participants in Brazil were reviewed and approved by the Tropical Medicine Research Center (CAAEs: 0008.0.046.000–11, 0449.0.203.000–09) and the Ethics Committee of the Federal University of Minas Gerais (CAAE: 27466214.0.0000.5149), Brazil. The human study in Cambodia was approved by the Institutional Review Board (IRB), National Institute of Allergy and Infectious Diseases (NIAD), National Institutes of Health (NIH), and National Ethics Committee for Human Research (NECHR), Cambodia (ClinicalTrials.gov Identifier: NCT00663546). Written informed consent was obtained from each participant. Written informed consent to participate in this study was provided by the participants’ legal guardian/next of kin.

### Study population, clinical samples and ethical statements

The study population included 121 subjects living in the Brazilian Amazon and diagnosed with vivax malaria at the Research Center for Tropical Medicine (CEPEM) in Porto Velho, Rondônia, Brazil, in the years of 2014 (69 serum samples) and 2019 (52 serum samples and 22 PBMC samples) during the transmission season. The inclusion criteria in this study included acute illness at the time of blood sampling and presence of *P*. *vivax* parasites assessed by blood smear microscopy. Serum samples from 15 healthy donors living in the same area were collected and defined as the “malaria-exposed” experimental group. Healthy subjects (N = 5) from Belo Horizonte, Minas Gerais State, Brazil, a non-endemic area for malaria, were also recruited as malaria-naïve controls (2019–2021). This study was approved by the Research Ethics Council of the Federal University of Minas Gerais, Brazil (CAAE 27466214.0.0000.5149). For some experiments, samples collected in Brazil were frozen and transported on dry ice to the U.S. National Institutes of Health (NIH), Bethesda MD, USA.

Sera collected from 55 *P*. *vivax* infected subjects (3–55 years old) in Pursat, Cambodia, between 2008 and 2018, under a protocol approved by NIAID and the National Ethics Committee on Human Research (ClinicalTrials.gov NCT00663546), were used to perform IgG ELISA assays against *Pv*Vir14. Sera from 28 Malian adults (range of age: 18–55 years old) collected between 2015 and 2019 were used to investigate reactivity to *Pv*Vir14 and were approved for use under a protocol approved by the NIAID IRB and the University of Bamako (FMPOS) Ethical Review Committee, and the study registered at ClinicalTrials.gov (NCT02334462).

An additional malaria-naive control group included healthy adult subjects (N = 5) who donated samples during participation in clinical studies at the U.S in 2020. NIH (Bethesda, MD).

Each volunteer was required to sign a written informed consent specific to this study, and blood was obtained upon receiving the said document.

### Expression and purification of *Pv*Vir14, *Pv*DBP-RII, *Pv*CSP and *Pvs*230D1

The full-length (325 amino acids) predicted *Pv*Vir14 protein was expressed in *Escherichia coli*. Briefly, the recombinant plasmid with the PUC/*Pv*Vir14 synthetic gene (GenScript) was resuspended and transformed with competent cells from *E*. *coli* XL-1 Blue (Phoneutria, Brazil). Positive clones were confirmed through digestion using Xho1 and Nhe1 enzymes. The *Pv*Vir14 insert was then cloned into a bacterial expression vector (pET28aTEV) and transformed with competent *E*. *coli* BL21-star (Thermo Fisher Scientific, USA) by electroporation (Bio-Rad Laboratories, USA). To confirm gene insertion, a colony PCR was performed using T7 primers (Macrogen, South Korea). To produce at large scale, 1mM of isopropyl β-D-1-thiogalactopyranoside (IPTG) was added following an incubation period of 3h at 37°C at 180rpm. Cells were ruptured by a high-pressure homogenizer and soluble fractions were obtained by centrifugation. The recombinant protein was purified using Ni^2+^ affinity chromatography with HisTrap HP 5 mL column (GE Healthcare, USA) coupled to an ÄKTA Prime Plus system (GE Healthcare, USA). The purified *Pv*Vir14 protein, having 325 amino acids and predicted molecular weight of 37kDa, was separated by SDS-PAGE. *Pv*s230D1M (domain 1 from Sal-1 strain) and *Pv*CSP (VK210 allele) were expressed in *Pichia pastoris* following similar procedures previously described for *Pf*s230D1M and *Pf*CSP [18,19] and the details for their production will be reported elsewhere. *Pv*DBP-RII was expressed in *E*. *coli* BL-21 cells and refolded as previously described [20,21].

### Detection of IgG and IgM antibodies by enzyme-linked assay (ELISA)

The presence of *Pv*Vir14-IgG and IgM in serum was determined through a conventional enzyme-linked immunosorbent assay (ELISA) performed as follows: 96-well plates were coated with antigen (0.5μg/mL) in carbonate buffer overnight at 4°C. Wells were then blocked with 0.5M chloride, 1% Triton X-100 and 1% bovine serum albumin in PBS (1X) and incubated for 2 hours at room temperature (RT) followed by incubation with the primary antibody at a concentration of 1:500 for IgG and 1:100 for IgM (serum from infected subjects). After washing 4 times with PBS (1X) plus 0.05% Tween 20, a peroxidase HRP-conjugated secondary antibody was added at a concentration of 1:10000 (IgG) and 1:8000 (IgM) and incubated for 1 hour at RT with shaking. The plate was washed 4 more times with 1X PBS Tween (0.05%) before the developing reagent (3.3’, 5’5 –Tetramethylbenzidine (TMB), SeraCare) was added to each well. The reaction was stopped after 15 minutes at RT by the addition of a TMB stop solution (SeraCare) and the optical density was measured at 450nm.

ELISA assays to detect the IgG subclasses were performed as previously described [22]. Sera were diluted at 1:50 and evaluated for each IgG subclass using the following mouse anti-human monoclonal antibodies: IgG1 clone 8c/6-39; IgG2 clone HP-6014; IgG3 clone HP-6050; IgG4 clone HP-6025) (Sigma, St.Louis, MO) according to the manufacturer’s instructions. Monoclonal antibody binding was detected with TMB peroxide substrate solution (Thermo Fisher). The *cut-off* value for detection was determined by testing 5 different negative control sera from subjects never exposed to malaria from Bethesda MD, United States of America. The mean optical density value at 450 nm ±3SD (VersaMax Microplate Reader) for duplicate determinations in negative sera was used as the cut-off value for different subclasses.

### Flow cytometry analyses of PBMCs from *P*. *vivax* infected, treated, or exposed subjects

We examined whether cellular immune responses were related to *Pv*Vir-14 seroreactivity among *P*. *vivax*-infected or exposed individuals, using flow cytometry and a panel of markers for the main B and T cell subtypes. The population was stratified by exposure, infection and seroreactivity status: *Pv*Vir14+ (corresponding to those with acute infection and high IgG titers for *Pv*Vir14; n = 8); *Pv*Vir14- (infected subjects with titers below the level of detection for *Pv*Vir14; n = 7); and HD (healthy donors in non-endemic areas–USA, n = 5; Brazil, n = 5). PMBCs were thawed in a 37°C water bath, resuspended in complete RPMI, and then in PBS. Cells were stained with the following antibodies: CD3 FITC (clone SK7), CD4 PerCP-Cy5.5 (clone SK3), CD8 APC-Cy7 (clone SK1), CD14 AlexaFluor700 (clone HCD14), CD16 PE-Cy7 (clone 3G8), CD19 BV605 (clone HIB19), CD21 BV711 (clone B-ly4), CD27 APC (clone LG3A10), and CD56 BV785 (clone 5.1H11). All antibodies are from BioLegend, except for CD21 BV711 (clone) which is from BD Biosciences. Cells were also stained with Zombie UV fluorescent dye (BioLegend) to assess live vs. dead cells. Cells were incubated at 4°C protected from light for 20 minutes while staining and then washed with PBS and resuspended in FACS Buffer. Flow cytometry was performed on a LSRII instrument (BD Biosciences) and analysis was done using FlowJo v.10. Cells were first gated for singlets by FSC-H and FSC-A, followed by live cells (FSC-A, live/dead) and lymphocytes (SSC-A and FSC-A). Monocytes were gated out of total lymphocytes. B cells were thereafter gated as CD19^+^ followed by CD27 and CD21 to distinguish (i) classical memory B cells (CD27^+^ CD21^+^), (ii) activated memory B cells (CD27^+^ CD21^-^), (iii) atypical memory B cells (CD 27^-^ CD21^-^), (iv) naïve B cells (CD27^-^ CD21^+^). T cells (CD3^+^) were gated out of total lymphocytes and thereafter as CD4^+^, CD8^+^ (CD3^+^CD56^-^) and NKT subsets (CD19^-^CD3^+^CD56^+^) (S1 Fig). Data analysis was followed by dimensionality reduction and visualization by t-Distributed Stochastic Neighbor Embedding (tSNE) or Principal Component Analysis (PCA) using Cytofikit [23].

### VDJ sequencing of memory B cells

Amplification of BCR heavy and light chains from single sorted B cells was performed by iRepertoire Inc. (Huntsville, AL, USA) as previously described [24]. Briefly, RT-PCR1 was performed with nested, multiplex primers covering both heavy, kappa, and lambda loci, and including partial Illumina adaptors. After RT-PCR1, the first round PCR1 products were rescued using SPRISelect Beads (Beckman Coulter, Brea, USA). A second PCR was performed with dual-indexed primers that complete the sequencing adaptors introduced during RT-PCR1 and provide plate positional information for the sequenced products. Sequencing was performed using the Illumina MiSeq v2 500-cycle kit with 250 paired-end reads.

### Statistical analysis

The Reactivity Index (RI), used to define positivity in serology assessments, was calculated by dividing the mean OD of the assessed subjects by the mean OD plus 3 standard deviations (SD) of the malaria-naïve control subjects (Confidence Interval of 99%). Values above 1 were considered positive. Samples were submitted to a normality test to define statistics. For non-normal data or non-equal variances among groups (3 or more groups), a Kruskal-Wallis test was performed. For normal data with equal variances, we performed ANOVA or, for comparison between 2 groups, T test. To determine equality of variances among normal populations, a Fisher’s exact test was performed, and considered significant when p<0.05.

## Results

### Epidemiological profile of selected subjects

The study population is composed of 121 *P*. *vivax-*infected adults from Porto Velho–Rondônia, Brazil and neighboring municipalities; 15 non-infected but exposed subjects from Porto Velho–Rondônia, Brazil; 5 non-infected and never exposed subjects from Belo Horizonte–Minas Gerais, Brazil; 55 *P*. *vivax-*infected adults and children from Pursat, Cambodia; 28 *P*. *falciparum-*infected adults from Bancoumana, Mali, West Africa; and 5 non-infected and never exposed subjects from Bethesda–Maryland, United States of America (Table 1). Concerning the infected Brazilian population, the majority were adults naturally exposed to malaria (*P*. *vivax* and/or *P*. *falciparum*) and the age range was 29–49 years. The sex ratio was 1:3 (female: 23.9%; male: 76.1%). The non-infected but currently exposed had an age range of 20–47 with a sex ratio of 2.6:1 (female: 72.22%; male: 27.77%). The non-infected/never exposed had an age range of 26–32, and a sex ratio of 1:1.5 (2 female, 3 male). For the Cambodian population, the range age was 3–55, and a sex ratio of 1:1.75 (female: 36.36; male: 63.63%). As for the Malian population, the age range was 18–55 with a sex ratio of 1:3.4 (female: 22.8%; male: 77.2%). Finally, the American population was all males with age range 49–53 years, and the bias was merely due to sample availability. For the endemic regions, considering that the main factor to be enrolled was an acute infection, we observed a clear male bias within infected populations, which is a common feature for vertebrates [25]. Among the sexual differences that can cause such bias are hormones, immunity, and exposure. The latter is well-known in malaria, as vector-borne parasitic infections are intrinsically related to the complex and active role of the vectors plus host behavior traits.

**Table 1:** Epidemiological and demographic characteristics of the subjects selected for the study.

**Epidemiological and demographic characterization**	
**Brazil: *P*. *vivax-*infected subjects (n = 121)**	
Age range, years	29–49
Sex, female:male	1:3
**Brazil: *P*. *vivax* non-infected but exposed subjects (n = 15)**	
Age range, years	20–47
Sex, female:male	2.6:1
**Brazil: malaria-naive subjects (n = 5)**	
Age range, years	26–32
Sex, female:male	1:1.5
**Mali (n = 28)**	
Age range, years	18–55
Sex, female:male	1:3
**Cambodia (n = 55)**	
Age range, years	3–55
Sex, female:male	1:1.75
**United States of America: malaria-naïve subjects (n = 5)**	
Age range, years	49–53
Sex, female:male	0:1

### IgG response to *Pv*Vir14 among subjects infected with *Plasmodium ssp*. and *P*.*vivax e*xposed without acute infection

**Fig 1:**
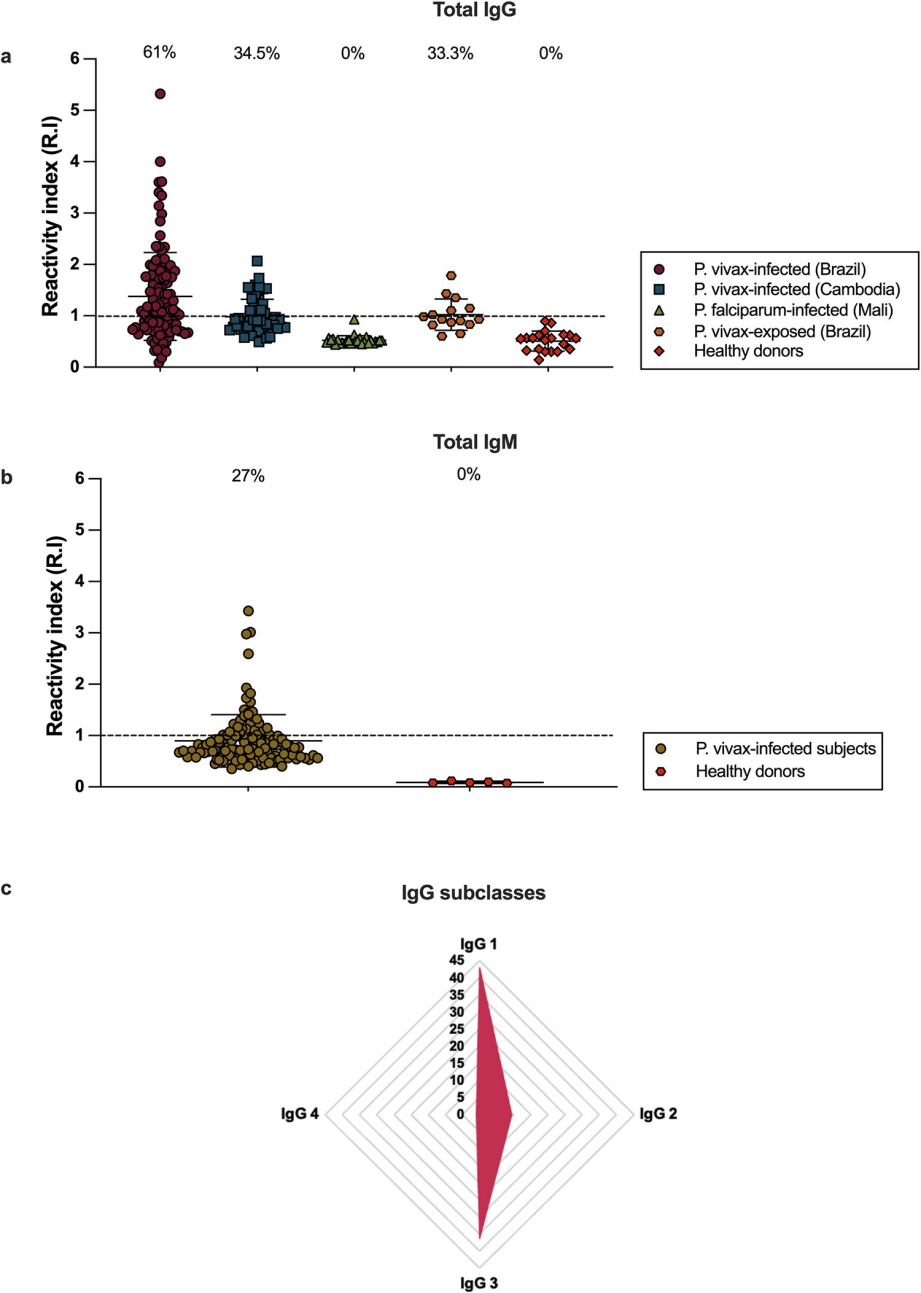
Relative quantity of anti-*Pv*Vir14 IgG and IgM antibodies, and prevalence of IgG subclasses to *Pv*Vir14. a) Subjects from the Brazilian Amazon (n = 121, Porto Velho–RO) and from Cambodia (n = 55) with an acute infection by *P*. *vivax—IgG titers*; Subjects from Western Africa (Mali) with an acute infection by *P*. *falciparum* (n = 28)–IgG titers; and subjects living in the Brazilian amazon (Porto Velho—RO), thus exposed to *P*. *vivax* but not infected at the moment of collection (n = 15). b) Subjects from the Brazilian Amazon (Porto Velho–RO) with an acute infection by *P*. *vivax* (n = 119)–IgM titers. For a and b, Y axis represents the mean reactivity index (RI) and the dotted line shows the seropositivity threshold (RI = 1). c) Frequency of detectable IgG subclasses against *Pv*Vir14 among infected Brazilian subjects. The radar charts are divided into nine lines, each of them representing a 5% value, data are presented (orange rhomboid) as the percentage of individuals (n = 121) with detectable levels for the four IgG subclasses against *Pv*Vir14.

Among 121 *P*. *vivax-*infected subjects in Brazil, 74 (61%) had detectable IgG against *Pv*Vir14, compared to 19 out of 55 (34.5%) Cambodian subjects. To confirm that *Pv*Vir14 is a specific and exclusive target of *P*. *vivax*, sera from 28 *P*. *falciparum* infected subjects from Mali (Western Africa) were tested; *P*. *vivax* infections have not been reported at the study site in Mali. Of 28 sera tested, none were reactive to *Pv*Vir14, consistent with their lack of prior exposure to *P*. *vivax* and the absence of a *P*. *falciparum* ortholog for this protein. To assess whether *Pv*Vir14 seroreactivity is present even without an acute infection, we assayed sera collected from 15 uninfected subjects living in *P*. *vivax* endemic areas for anti *Pv*Vir14-IgG levels. Five subjects (33%) had detectable antibody titers three of the five seroreactive had a known history of prior clinical malaria, which might explain the presence of circulating antibodies, while the malaria history for the other two subjects is unknown (Fig 1A).

### Anti-*Pv*Vir14 IgM is detected in *P*. *vivax*-infected individuals

We investigated IgM antibodies targeting *Pv*Vir14. During *P*. *vivax* acute infection, 32 out of 119 Brazilian subjects (26.9%) displayed *Pv*Vir14-IgM antibodies in their sera (Fig 1B); samples from 2 of the 121 subjects had been exhausted before IgM testing.

### IgG1 and IgG3 responses are dominant in response to *Pv*Vir14

The functionality of antimalarial IgG antibodies may depend on their subclass [26]. We assessed IgG1, IgG2, IgG3 and IgG4 levels within the *P*. *vivax*-infected individuals from Rondônia, Brazil. Detectable IgG1 (42.85%) and IgG3 levels (36.13%) were most frequent, while IgG2 (frequency of 9.24%) was less frequent and IgG4 (0.84%) was detected in a single individual (Fig 1C).

### *Pv*Vir14-IgG titers correlate to other *P*. *vivax* protein-IgG titers

**Fig 2:**
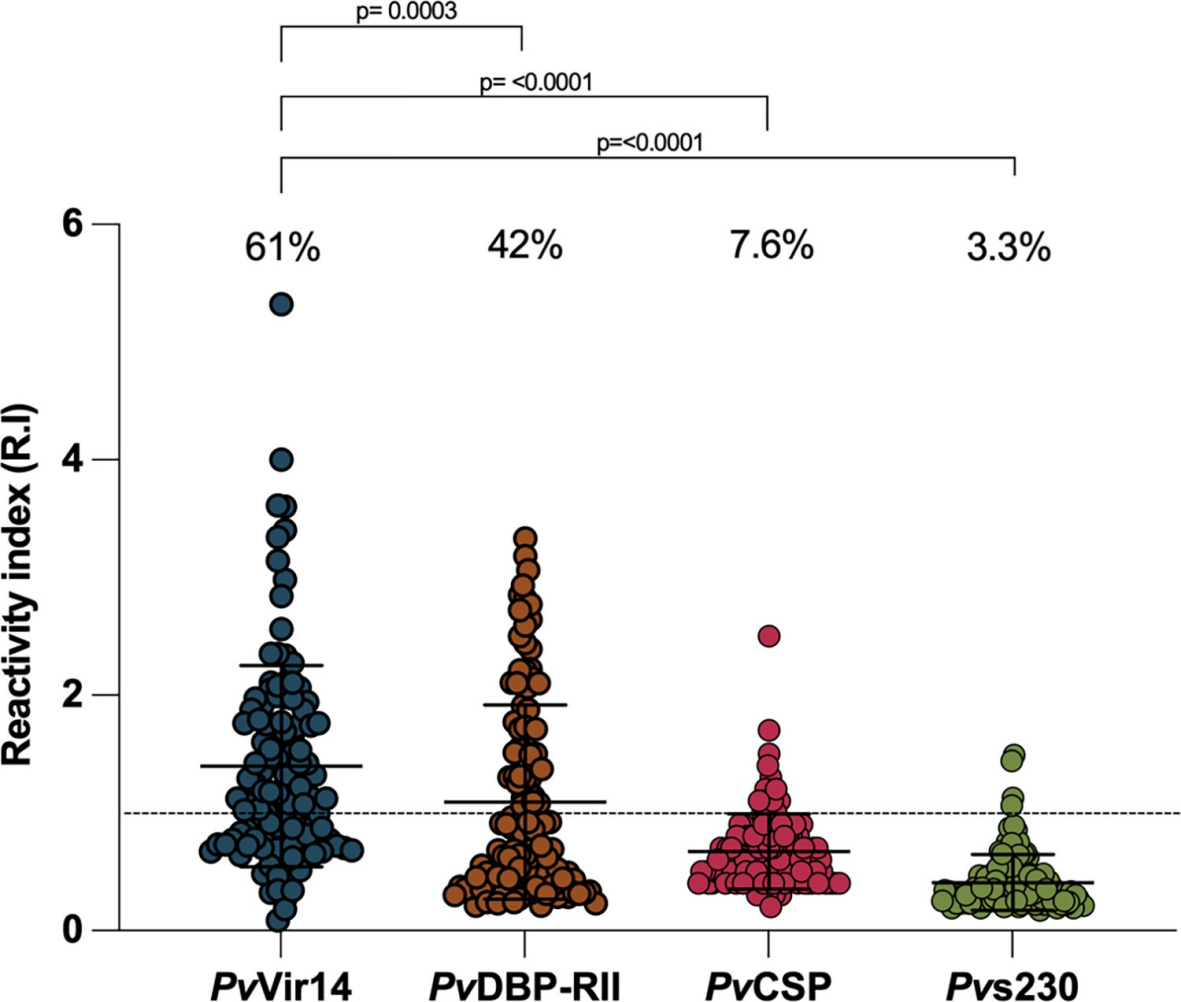
Seroreactivity to *Pv*Vir14 versus other *P*. *vivax* candidate vaccine antigens (*Pv*CSP, *Pv*DBP-RII and *Pv*s230D1). (a) IgG Reactivity Index against *P*. *vivax* antigens in *P*. *vivax* infected subjects. For (b-d), mean OD of anti-*Pv*Vir14 IgG versuis (b) mean OD of anti-*Pv*CSP, (c) mean OD of anti-*Pv*DBP-RII (d) mean OD of anti-*Pv*s230D1. The dotted line marks the minimum value of the mean OD for a subject to be considered above the level of detection (> 1, R.I) Data were analyzed considering a 99% confidence interval (CI). P < 0.05 was considered significant (* p<0.05; **p<0.01; ***p<0.001).

We compared IgG reactivity to *Pv*Vir14 with IgG reactivity to other *P*. *vivax* proteins, including well-characterized vaccine candidates from sporozoite/liver (*Pv*CSP), blood (*Pv*DBP-RII) and mosquito-sexual stages (Pvs230D1). In ELISA assays to measure total IgG, the positivity rates were as follows: 61% for *Pv*Vir14, 7.6% for *Pv*CSP, 42% for *Pv*DBP-RII and 3.3% for *Pv*s230 domain 1 (Fig 2A). The frequency of seroreactivity during infection was significantly higher for *Pv*Vir14 compared to the three proteins analyzed here (*Pv*CSP: <0.0001, *Pv*DBP-RII: 0.0003, *Pv*s230: <0.0001, Fisher’s exact test). In addition, a statistically significant but weak correlation between *Pv*Vir14-IgG levels and IgG levels to the sporozoite/liver and blood stage proteins was observed (*Pv*CSP, p = 0.0077; r = 0.2442 and *Pv*DPB, p = 0.0042; r = 0.2614, Spearman rank test) (Fig 2B and 2C), but not to the sexual stage Pfs230D1 protein (Fig 2D).

### Proportion of atypical B cells (AtMBCs) differ based on *P*. *vivax* infection

**Fig 3:**
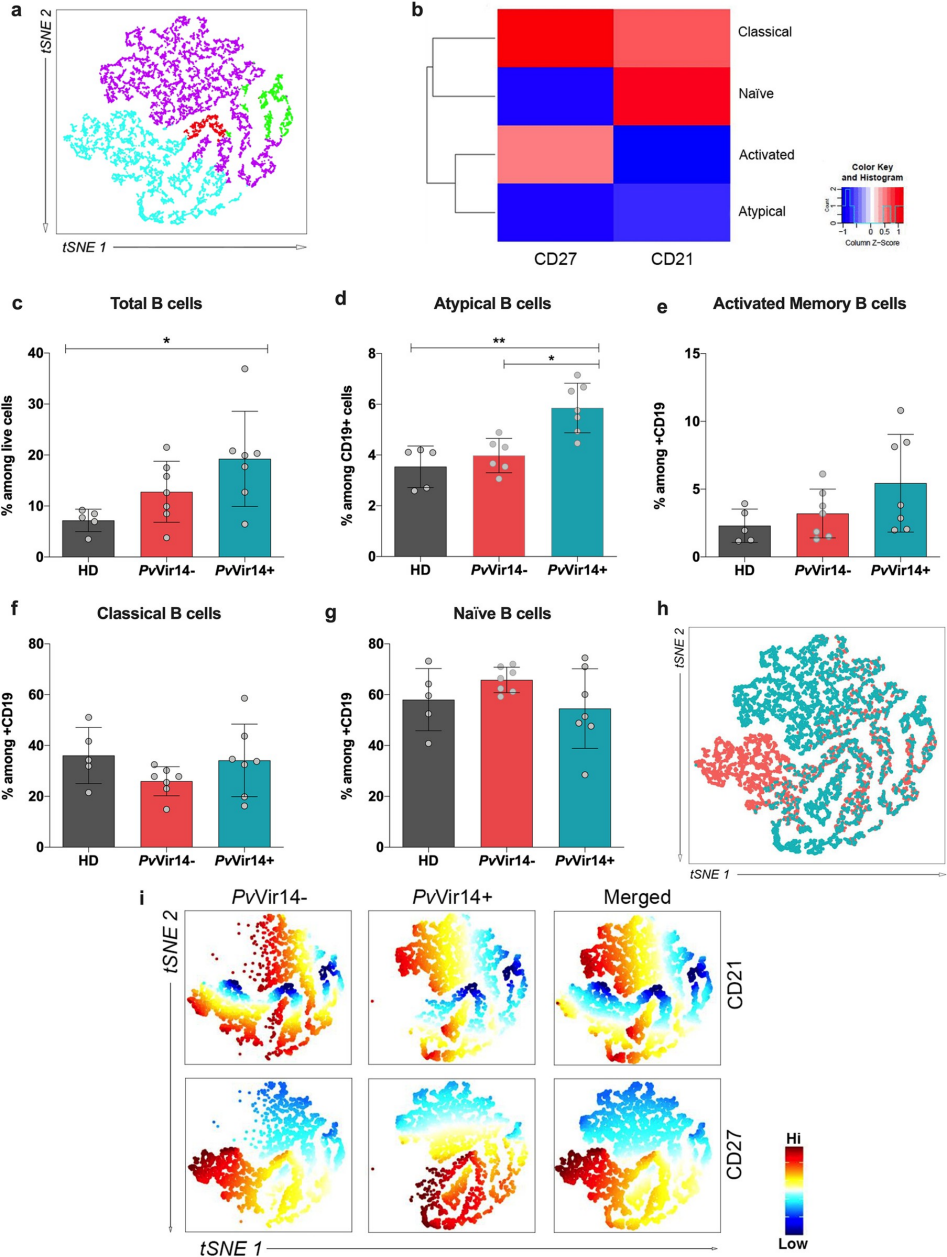
B cell phenotypic analysis by multiparametric flow cytometry. (a) Unsupervised high dimensional analysis of flow cytometry data (tSNE) from combined data from all B cells populations. Activated (red), Atypical (green), Classical (light blue), and Naïve (purple). (b) Heatmap showing marker expression within each cluster previously identified among B cells. (c) Bar graphs shows percentage of CD19^+^ (d), Atypical Memory B cells CD19^+^CD27^-^CD21^-^ (e), Activated Memory B cells CD19^+^CD27^+^CD21^-^ (f), Classical Memory B cells CD19^+^CD27^+^CD21^+^ (g) and Naïve B cells CD19^+^CD27^-^CD21^+^ from total Live PBMC cells. (h) Unsupervised high dimensional analysis of flow cytometry data (tSNE) from combined data of acute infected subjects with or without antibody titers against *Pv*Vir14 gated on LiveCD19^+^.The darker the color (reddish), the greater the intensity of expression. (i) Diffusion map of CD21 (top) or CD27 (bottom) intensity of expression depicting each group (*Pv*Vir14+: n = 7, *Pv*Vir14-: n = 7): and merged plot. P < 0.05 was considered significant (* p<0.05; **p<0.01; ***p<0.001).

B cells were clustered in 4 groups: Classical, Naïve, Activated, and Atypical (Fig 3A) based on levels of CD21 and CD27 expression (Fig 3B). Total B cells (as a proportion of live cells) were elevated in infected Brazilian subjects when compared to malaria-naïve healthy U.S. donors (Fig 3C), with the highest proportions seen in the *Pv*Vir14+ group (p = 0.022, One-way ANOVA). Among the B cell subpopulations, AtMBCs (as a proportion of total B cells) were higher for the *Pv*Vir14+ subjects in comparison to the other two groups (*Pv*Vir14- vs *Pv*Vir14+, p = 0.028; HD vs *Pv*Vir14+, p = 0.0039; Kruskal-Wallis with Dunn’s multiple comparison test) (Fig 3D), suggesting a relationship between *Pv*Vir14 seroreactivity and AtMBCs. Proportions of other B cell subsets did not differ significantly on the basis of *Pv*Vir14 seroreactivity, although there were trends for activated memory B cells to increase in the *Pv*Vir14+ group and for naïve B cells to increase in the *Pv*Vir14- group (Fig 3E–3G). These latter trends were also observed through the t-Distributed Stochastic Neighbor Embedding (tSNE) analysis, which demonstrated higher intensity of CD21 in *Pv*Vir14+ group while *Pv*Vir14- showed higher intensity of CD27 (arrows) (Fig 3H and 3I)

### CD4+ T cell, CD8+ T cell, and NKT cell frequencies vary with *Pv*Vir14 seroreactivity

**Fig 4:**
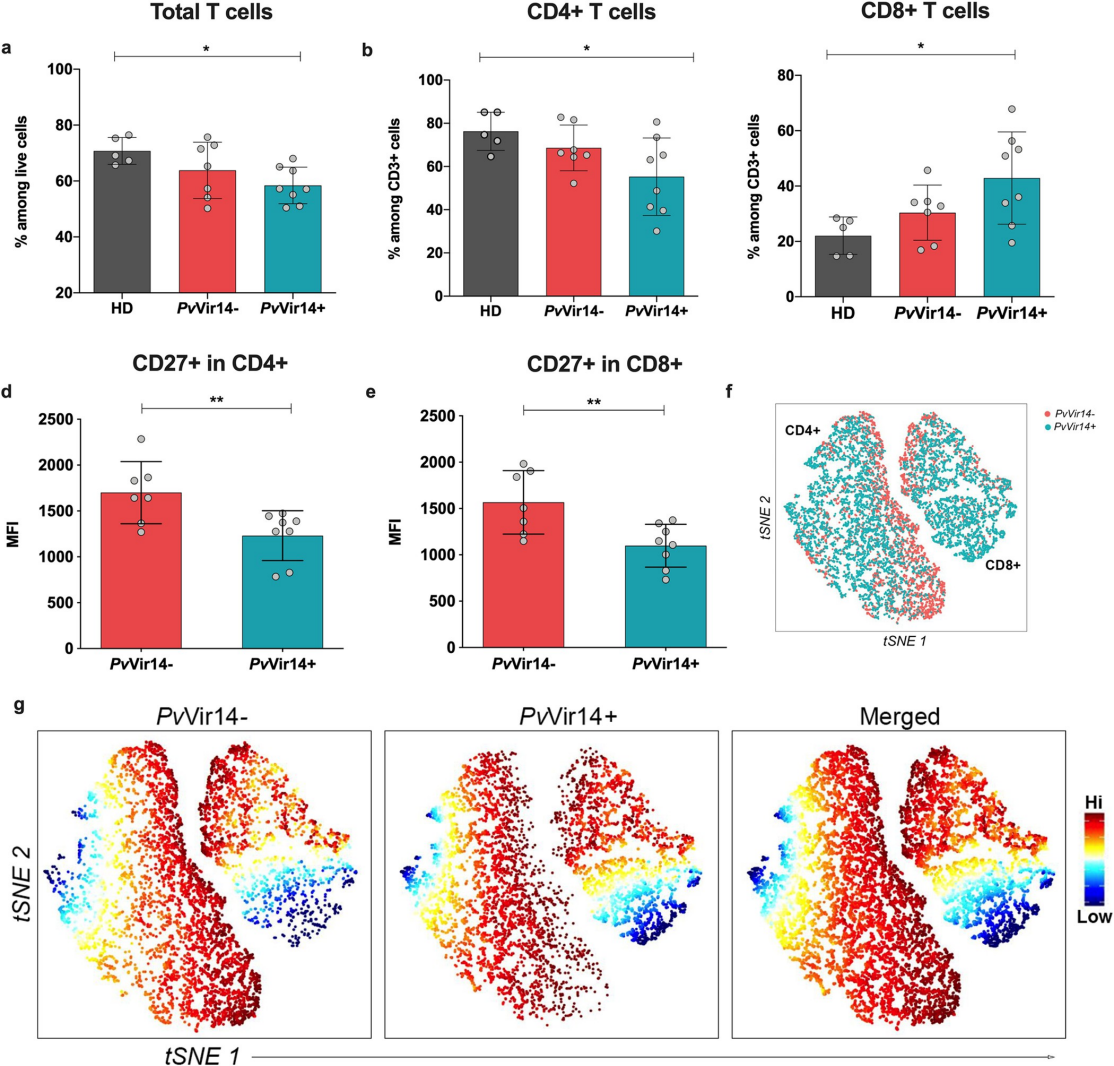
T cell phenotypic analysis by multiparametric flow cytometry. Bar graphs show percentage of CD3^+^ T cells (a) CD3+CD56^-^CD4^+^ T cells (b) and CD3^+^CD56^-^CD8^+^ T cells (c) from total Live PBMCs. Bar graphs show Mean Intensity Fluorescence (MFI) of CD27^+^ in CD4^+^ (d) or CD8^+^ (e). (f) tSNE from combined data of *Pv*Vir14^+^ (green) or−(red) subjects gated on LiveCD3^+^CD56^-^ T cells. (g) Expression map of CD27^+^ cells from F depicting PvVir14- (left), *Pv*Vir14+ (middle) and Merged (right). The increasing intensity of red color indicates higher CD27 signal, and the increasing intensity of blue color indicates decreasing CD27 signal. Grey bars represent Healthy Donors (HD), red bars represent PvVir14- and green bars represent *Pv*Vir14+ subjects. P <0.05 was considered significant (* p<0.05; **p<0.01; ***p<0.001).

T cells can re-distribute and levels can fluctuate during malaria infections [27–29]. In our *P*. *vivax-*infected participants, total T cell levels (as a percentage of all live cells) were lower than those of uninfected U.S. donors, but this difference was only statistically significant for the *Pv*Vir14+ group (p = 0.030, One-Way ANOVA) (Fig 4A). When measured as a proportion of total CD3+ T cells, CD4^+^ T cells were lower and CD8^+^ T cells were higher in the *Pv*Vir14+ group (Fig 4A and 4C). Based on Mean Fluorescence Intensity (MFI), CD27^+^ expression level was signficantly lower in *Pv*Vir14+ versus *Pv*Vir14- individuals for both CD4^+^ and CD8+ (p = 0.010 and p = 0.007 respectively, T test) (Fig 4D and 4E). When using a t-stochastic neighbor embedding (tSNE) to reduce dimensionality, the data analysis resulted in the identification of 2 clusters corresponding to CD4^+^ and CD8^+^ T cells (Fig 4F) and a closer examination at the single-cell level confirmed that the *Pv*Vir14- group indeed is expressing a higher intensity of CD27^+^ on CD4 and CD8 T cells than *Pv*Vir14+ (Fig 4G).

**Fig 5:**
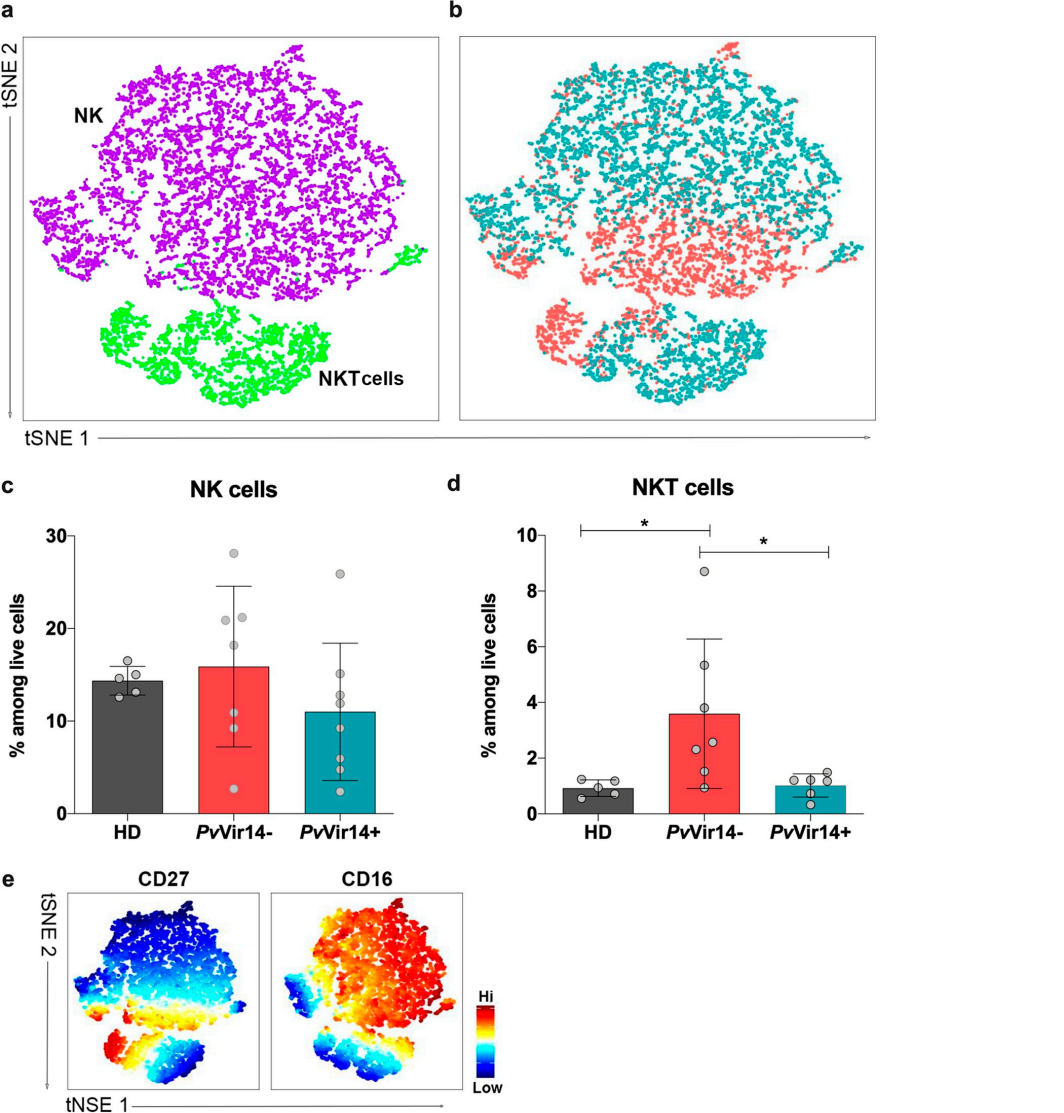
NK and NKT cell population dynamics by multiparametric flow cytometry. (a) tSNE from combined data of *Pv*Vir14^+^ or *Pv*Vir14^–^subjects gated on CD19^-^CD14^-^CD56^+^ NK (purple) and NKT cells (green). (b) tSNE from (a) depicting *Pv*Vir14+ (red) and *Pv*Vir14- (blue) cells. (c) NK cells (CD3^-^CD19^-^CD14^-^CD56^+^) as a percentage of live cells, by group. (d) NKT cells as a percentage of live cells, by group. (e and f) Expression levels of distinguishing markers of NK and NKT cells. P <0.05 was considered significant (* p<0.05; **p<0.01; ***p<0.001).

As Natural Killer (NK) cells are known to be one of the first innate cells that respond to malaria parasites [30], helping activate adaptive responses, its population was also assessed. NK and Natural Killer T (NKT) cells were defined as CD3^-^CD19^-^CD14^-^CD56^+^ and CD3^+^CD56^+^, respectively, and constituted 2 clusters (Fig 5A). Measured as a proportion of all live cells, NK cells did not differ significantly between groups (Fig 5B), whereas NKT cells were significantly higher in the *Pv*Vir14- group than *Pv*Vir14+ group (p = 0.041, t test) (Fig 5C). Of interest, subjects who lack circulating antibodies against *Pv*VIR14 have higher expression of CD27^+^ on cells when compared to those presenting serological reactivity to the protein; no difference was observed regarding CD16^+^ (Fig 5E).

### B cell subsets, *Pv*Vir14 circulating antibodies, and NKT population decline in brazilian subjects after drug treatment

**Fig 6:**
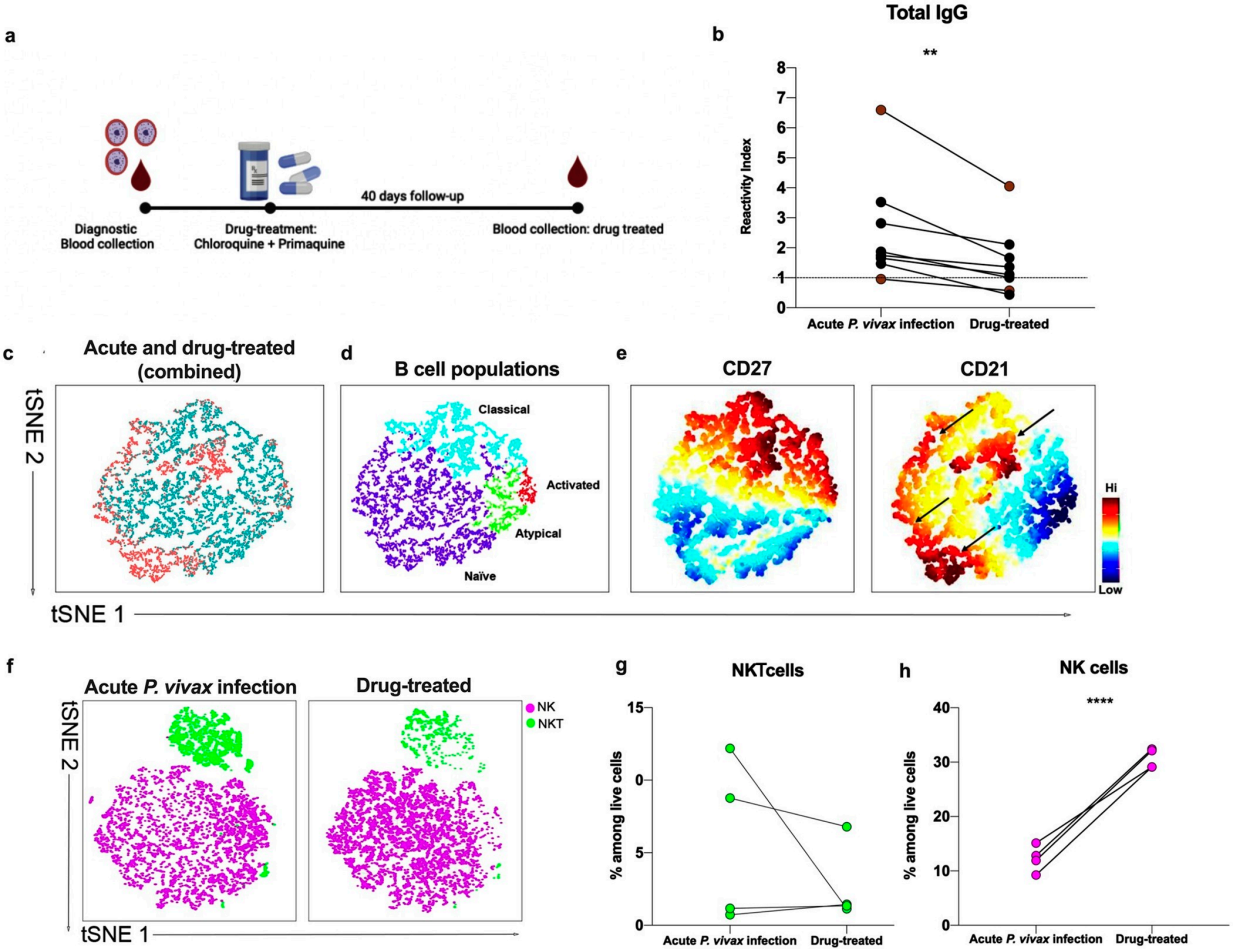
Drug-treated subjects’ profile for antibody titers, B cells, and NK and NKT cells. (a) Sample collection and therapeutic scheme. Created with BioRender.com. (b) Total IgG levels from two different points in time: acute infection and post-drug treatment. Serum samples were paired and belonged to the same subjects (acute and post-treatment samples). Two subjects, represented in purple, were also infected at the follow-up 40 days after primary acute infection. (c) tSNE from combined data of *Pv*Vir14+ during acute (red) or post treatment (blue) gated on Live CD19^+^ B cells. (d) tSNE from (c) depicting B cell populations Atypical Memory B cells CD19^+^CD27^-^CD21^-^ (green), Activated Memory B cells CD19^+^CD27^+^CD21^-^ (red), Classical Memory B cells CD19^+^CD27^+^CD21^+^ (light blue) and Naïve B cells CD19^+^CD27^-^CD21^+^ (purple). (e) Expression plot of distinguish markers CD27 (left) and CD21 (right). The arrows indicate populations with higher CD21 expression. (f) tSNE from combined data of *Pv*Vir14+ during acute (left plot) or post treatment (right plot) gated on LiveCD19^-^CD14^-^CD56^+^ showing NK CD3^-^CD56^+^ (purple) and NKT CD3^+^CD56^+^ (green) cells. (g) Percentage of NKT cells among live cells in Acutely infected and Drug-treated subjects. (h) Percentage of NK cells among live cells in Acutely infected and Drug-treated subjects. The data were analyzed considering a 99% confidence interval (CI). P < 0.05 was considered significant. (* p<0.05; **p<0.01; ***p<0.001).

Upon the diagnosis of acute malaria, all subjects in this study received treatment consisting of Primaquine and Chloroquine (Fig 6A). Forty days after starting the treatment, serum *Pv*Vir14-IgG levels had significantly decreased among 8 individuals who returned for a follow up visit (p = 0.0078, Wilcoxon matched-pairs) (Fig 6B). Two subjects (beige dots) presented with parasitemia at the time of the post-treatment follow-up. Since *P*. *vivax* is known to cause relapses frequently, the parasitemia at follow up may represent a new infection, a relapse of liver hypnozoites present at the time of first diagnosis, or a recrudescence of blood-stage parasites that were not cleared at the first treatment. The two subjects presenting with infection at the follow up visit were treated according to the therapeutic scheme in Brazil (Primaquine + Artemether-Lumefantrine).

Among the 8 returning subjects, immune cell samples from 4 were available for phenotypical characterization followed by tSNE and clustering analysis. The distribution of cells in the tSNE plots differed at the time of acute infection versus follow up (Fig 6D). Following antimalarial treatment, CD21^+^ expression decreased in naïve and classical B cell subsets (Fig 6E and 6F).

In addition, although there is visual suggestion of a decrease in NKT cells in drug-treated subjects, the difference is not observed when assessed as a percentage among live cells (Fig 6G). Levels of NK cells were significantly increased as observed in the percentage of live cells (p = <0.0001, T test) in the drug-treated group (Fig 6H). Critically, the results should be seen as a trend, since the sample size of this group was low when compared to the initial number of patients enrolled.

### Memory B cell V gene usage and CDR3 length during *P*. *vivax* infection

**Fig 7:**
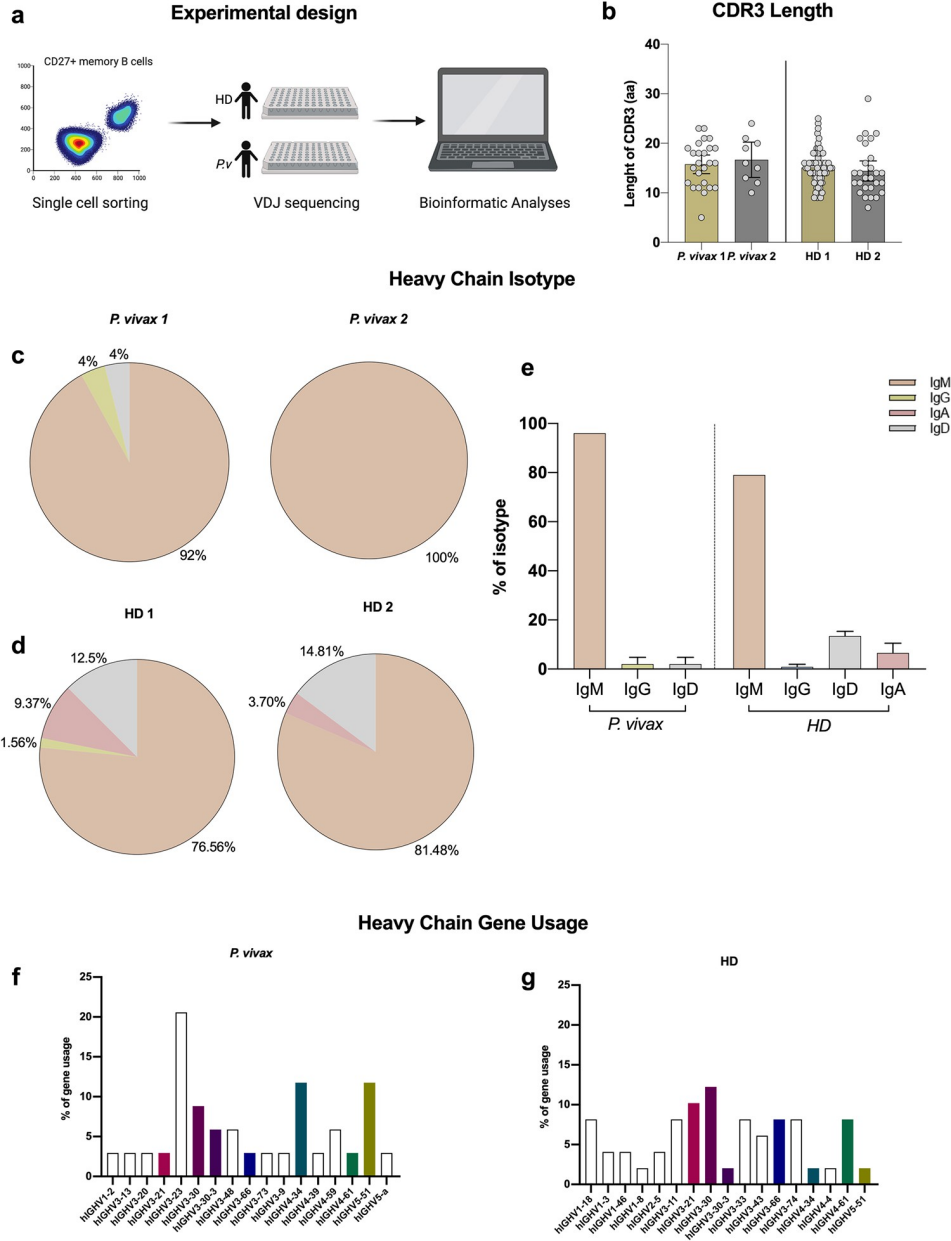
B cell receptors: CDR3 length, isotypes and V gene usage. (a) Experimental design of the sorting process. Single CD27+ memory B cells of two *P*.*vivax*-infected subjects with the highest anti-PvVir14+ IgG titers, and two malaria-naïve U.S. subjects, were sorted into a 96-well plates and had V gene usage analyzed. Created with BioRender.com. (b) Length of CDR3 sequences of two *P*. *vivax*-infected subjects (*P*. *vivax* 1 and *P*. *vivax* 2) from the Brazilian Amazon and two healthy donors (HD1 and HD2) from the U.S. The y axis represents the absolute number of amino acids in individual B cells as well as the mean for all BCR sequenced from each subject. (c) Heavy chain isotype of BCR sequenced from two *P*.*vivax*-infected subjects, presented in pie charts as proportions. (d) Heavy chain isotypes of BCR sequenced from two HD donors, presented in pie charts as proportions. (e) Heavy chain isotyping of all four subjects analyzed (two *P*. *vivax*-infected and two HD). The y axis represents the percentage of BCR belonging to specific antibody classes. (f) Heavy chain gene usage of BCR sequenced for two *P*. *vivax*-infected subjects. (g) Heavy chain gene usage of BCR sequenced for two HD subjects. The y axis presents the frequency of individual V genes as a percentage of all BCR sequenced in the two group. Open bars indicate V genes unique to a group; colored bars indicate V genes shared between the groups, and the colors correspond to the same V gene in each group.

To further characterize the B cell response in *P*. *vivax* infected subjects, single memory B cells were sorted from PBMCs of (i) two subjects with acute infection and high titers against *Pv*Vir14 from the Brazilian Amazon; (ii) two non-infected subjects from the USA (Fig 7A). All four subjects had their V fragment (of its VDJ genes) and CDR3 region amplified using RT-PCR. When comparing lengths of CDR3, the major site for repertoire variation, *P*. *vivax*-infected subjects and healthy donors did not differ (USA) (Fig 7B). As for the heavy chain isotype, IgM was most commonly detected for all subjects, particularly for *P*. *vivax*-infected individuals, albeit group differences were not significant in this small sample size (Fig 7C–7E). Concerning heavy chain gene usage, 17 genes from *P*.*vivax*-infected subjects and 17 genes from non-infected subjects (HD) were identified. Seven genes were shared between the two groups: hIGHV3-21, hIGHV3-30, hIGHV3-33, hIGHV3-66, hIGHV4-34, hIGHV4-61, hIGHV5-51 (Fig 7F and 7G). One V gene unique to the infected individuals–hIGHV3-23 –comprised 20% of gene usage for those two subjects. The gene hIGHV4-59, already found in other malaria-infected populations around the world [31], was also detected in the infected but not uninfected donors.

## Discussion

*P*. *vivax* has attracted increasing attention as the awareness of severe and lethal cases increased [29], and this parasite accounts for ~13% of the cases in Africa and >70% of cases in Asia and in the Americas [1]. Nevertheless, *P*. *vivax* receives little attention when compared to *P*. *falciparum*. While vaccines could be a valuable tool for control of *P*. *vivax* [32] when used with other measures such as timely diagnosis and access to treatment [33], only a few *P*. *vivax* candidates are in clinical and pre-clinical phases (including *Pv*DBPII, *Pv*DBPII/GLA-SE, *P*. *vivax* irradiated sporozoites, *Pv*CSP-derived long synthetic peptides, VMP001-AS01B and *Pv*s25) [30].

In the present study, a member of a *P*. *vivax* variant antigen family (Vir) was detected in patients’ urine and was assessed for seroreactivity and related immunological profiles. Pathogen proteins detected in body fluids during infection are widely used as biomarkers of different infections [34]. Although it is well known that Vir proteins are extremely diverse, their functions and localizations are mainly unknown [16]. The fact that Vir is a superfamily carrying genes that differ greatly in size and number of exons [35] indicates that the proteins within the families might differ in function and, consequently, in immunogenicity.

Here, we observed that the prevalence of *Pv*Vir14 IgG antibodies during acute infection was 61% in Brazil and 34.5% in Cambodia. The greater prevalence in the Brazilian amazon population is probably due the different transmission setting when compared to Cambodia. While Brazil is known to have a high API (Annual Parasite Index) in endemic regions such as Rondonia [36], prevalence of this parasite in Cambodia has decreased in the last decade [37]. Another fact worth mentioning when discussing the antibody prevalence among those two different populations is that the Cambodian one comprises subjects under the age of 5 (age range 3–55), while the youngest Brazilian subject is 29 years old. It is well known that humoral immunity against malaria is dependent on age and exposure, but the cumulative number of episodes is also a main factor [38]. A serological study assessing other Vir proteins in Brazilian subjects found a frequency of detectable IgG of 26% [39], a lower prevalence than that observed in our study. Notably, when assessed in a *P*. *falciparum*-infected population in high-transmission Mali that is not exposed to *P*. *vivax*, none of the subjects recognized the protein, confirming that *Pv*Vir14 responses are exclusive to *P*. *vivax*. Besides IgG, a significant percentage (27%) of *P*. *vivax*-infected subjects from Brazil also displayed IgM antibodies against *Pv*Vir14. Although the IgM role in protective immunity against malaria is still unclear, IgM to the surface of merozoites inhibits binding in a complement-dependent manner and reduces the odds of clinical malaria [38]. Nonetheless, the role of IgM responses may vary according to the antigen, parasite life stage and host genotype [40,41]. While some studies in *falciparum* malaria have shown that IgM levels decline shortly after acute infection [42], others suggested long-term maintenance of IgM following clinical malaria [41,43].

In addition to IgM, cytophilic IgG subclasses can also work through complement fixation for some antigens and have been associated with protection against malaria [44,45]. In the present study, the two cytophilic antibody subclasses were present in 42.85% (IgG 1) and 36.13% (IgG 3) of the acutely infected subjects. In general, these antibodies may have a critical role in the development of premunition [46], and while their presence does not guarantee protection against clinical malaria, they may limit further complications [47].

When compared to other well-known *P*. *vivax* proteins from different phases of the parasite’s life cycle, *Pv*Vir14 showed more frequent seroreactivity. It has been described that vir genes from different families might be profusely expressed in different isolates at noun unspecified moments, unlike proteins such as *Pv*DBP or *Pv*CSP that are known to be expressed at a specific phase of the cycle [48]. The fact that those proteins are being expressed in an abundant and constant way could be the reason why antibody levels are being detected in a higher frequency. Nevertheless, seroreactivity to *Pv*VIR14 positively correlated with that to *Pv*CSP (liver stage) and *Pv*DBP-RII (blood-stage). Although the functional role of anti-*Pv*Vir14 has yet to be defined, the prevalence of *Pv*Vir14-IgG is consistent with blood-stage parasite expression, which generally elicits a relatively greater antibody response [49,50].

Since *Pv*Vir14 is a novel protein with features that suggest potential as a diagnostic or therapeutic target, we characterized immune cell subsets among acutely infected subjects with or without anti-*Pv*Vir14 titers. Our results demonstrated an expanded population of atypical memory B (AtMBCs) lacking CD21 and CD27 expression. The presence of this subset has been extensively described in subjects known to be chronically infected with malaria and living in varied transmission settings [51,52]. In our study, the subjects comprising the *Pv*Vir14+ group have a higher median of previous episodes (9.2 episodes/lifetime) than the subjects within the *Pv*Vir14- group (median: 7.8 episodes/lifetime), and such profile corroborates previous studies correlating AtMBC levels with past malaria infections [53]. Despite being a very common cell type among individuals exposed and/or infected with *Plasmodium*, this subset is also found in many other diseases, especially chronic ones such as HIV, hepatitis C (HBC), and lupus [54,55].

Whereas in broad terms AtMBCs are mainly defined by CD21^-^ CD27^-^, the presence/absence of several other expression factors distinguish AtMBC and its functions [56]. During infection, expanded AtMBC numbers have been thought to imply an impaired B cell response [57,58]. However, recent research finds that atypical B cells are part of an alternative B cell lineage that is a normal feature in healthy immune responses [59], and this may explain our finding that *Pv*Vir14 antibodies are related to an increase of AtMBCs. However, other studies suggested that a reduced expression of CD21 can be associated with complicated malaria [59,60], and thus may also be a feature of an impaired immune response.

When running a high dimensional analysis, classical and naïve B cells from the *Pv*Vir14+ subjects showed a higher intensity of CD21 whilst those from *Pv*Vir14- subjects showed a higher intensity of CD27. Although the frequency of these B cell subsets did not differ between the groups, the differential intensity of these markers may suggest that the cells are functioning differently. We speculate these differences may also contribute to inter-individual differences in *Pv*Vir14-IgG production. While B cells and antibody responses have a key role in protection, immunity to vivax malaria has recently been linked to the presence and functionality of T cells [25]. Assuming the importance of T cells in generating long-lasting protection, we assessed T cell subsets among infected subjects with or without anti-*Pv*Vir14 antibodies. We observed that CD4+ cells are lower and CD8+ cells are higher in *Pv*Vir14-IgG+ subjects compared to malaria-naïve healthy donors. A CD4+ decrease is a common profile for *P*. *vivax*-infected individuals, potentially due to a propensity for apoptosis, loss of proliferative capacity, “exhaustion”, or redistribution to sites of inflammation [61–63]. Whereas CD8+ T cells have not been thought to play a major immune role during blood-stage infection since red blood cells do not usually express human leucocyte antigen class I (HLA-I), it has been recently demonstrated that some reticulocytes (for which *P*. *vivax* has tropism) retain the protein translation machinery and surface-express these molecules [64,65]. In the same way as for B cells, *Pv*Vir14- subjects also demonstrated an upregulation of CD27 on both CD4^+^ and CD8^+^ T cells, indicating perhaps a larger number of activated T cells within this group [66] albeit the reason remains to be investigated.

Since treatment can alter the dynamics of the immune response, we explored immune response after Chloroquine/Primaquine therapy. IgG titers decreased for all assessed subjects after 40 days and anti-*Pv*Vir14 antibodies were still found circulating in the peripheral blood for most individuals (5/7 subjects) despite the marked reduction observed among the patients, consistent with studies showing that *Plasmodium* infections induce a patent but short-lived antibody-responses to blood-stage antigens [67–70].

As for circulating immune cells, classical and naïve B, NK, and NKT cells presented a different profile when comparing acute and post-treatment scenarios. During acute infection, both naïve and classical clusters presented a higher CD27 intensity of expression when comparing groups with or without anti-*Pv*Vir14 titers, but it is yet to be defined if the difference in expression has an impact on the disease progression [71]. Although two out of four subjects showed a major decrease in NKT percentage of cells the difference was not significant except when assessing expression levels of these cells, in which intensity decreased markedly when comparing acute and treated patients. As for NK cells, the difference between pre and post treatment was highly significant, with a marked increase. While the upregulated profile is common for acute patients, this state is usually transient and has been described before [72–74]. Nonetheless, NK activation and response can vary widely, especially related to individual genetic profiles which can determine expression levels and mediation factors such as activation through cytokines [75,76], therefore, further investigation regarding the subjects’ cytokine profile is warranted.

At the single B cell level, our study demonstrated that acute subjects present an unbalanced amount of *P*. *vivax*-IgM BCR versus other isotypes, indicating that those cells are probably engaged in the immune response to infection. Although predominance of IgM B cells has already been demonstrated for acute *P*. *falciparum* malaria subjects [77], little is known about the B cell biology of *P*. *vivax*. The IgM isotype has drawn attention lately since it can act as a protective factor through neutralization, opsonization, complement fixation, and functional inhibition of invasion with a higher and specific binding capacity [44,78]. At the gene level, we assessed the V region of the heavy chain segments known as V(D)J, which can undergo somatic hypermutation when challenged by an antigen [78], imbuing unique characteristics upon encounter with a particular pathogen. Among the genes unique to *P*.*vivax*-infected subjects, hIGHV3-23 was most common and accounted for 20% of all V gene usage within the heavy chain. The hIGHV3 family has already been related to high-affinity anti-*Plasmodium* antibodies against blood-stage antigens in naturally-infected subjects [79]. Of note, the hIGHV3-23 gene was recently identified as being present in children infected with *P*. *falciparum* for both *Pf* IgM^+^ and *Pf* IgG^+^, but with a lower percentage of use [77]. Unlike the latter, in our study, even though we selected subjects with high titers of anti-*Pv*Vir14, the cell sorting was not performed using antigen to isolate *Pv*-specific B cells, and this is a limitation of our study, as well as the number of subjects selected for the experiments. However, by sorting all memory B cells from donors, we were able to fairly compare between malaria-immune and malaria-naïve donors.

Our findings provide the first characterization of naturally acquired antibody responses from three different malaria endemic regions against a *P*. *vivax* protein that we find is secreted in the urine of malaria patients. Further studies are warranted to understand the role of *Pv*Vir14 in infections, and its potential as a target of control tools to help reduce malaria around the globe.

## Supporting information

S1 Fig Gating strategy of B, T and NK cell subsets. Cells were pre-gated for live/dead and then identified as follows: CD19^+^ B cells, activated memory B cells CD19^+^CD27^+^CD21^-^ (G1), Classical memory B cells CD19^+^CD27^+^CD21^+^ (G2), Naïve B cells CD19^+^CD27^-^CD21^+^ (G4), Atypical memory B cells CD19^+^CD27^-^CD21^-^ (G4), Live CD3^+^, NK cells (CD19^-^CD3^-^CD14^-^), NKT cells (CD19^-^CD3^+^CD56^+^), CD4^+^ and CD8^+^ T cells (CD3^+^CD56^-^).
(TIF)
