# Supplementary figures and images for: Immunological characterization of a VIR protein family member (VIR-14) in Plasmodium vivax-infected subjects from different epidemiological regions in Africa and South America
Source: PLoS Negl Trop Dis. 2023 Apr 7;17(4):e0011229. doi: 10.1371/journal.pntd.0011229 (PMC10115285; doi:10.1371/journal.pntd.0011229)

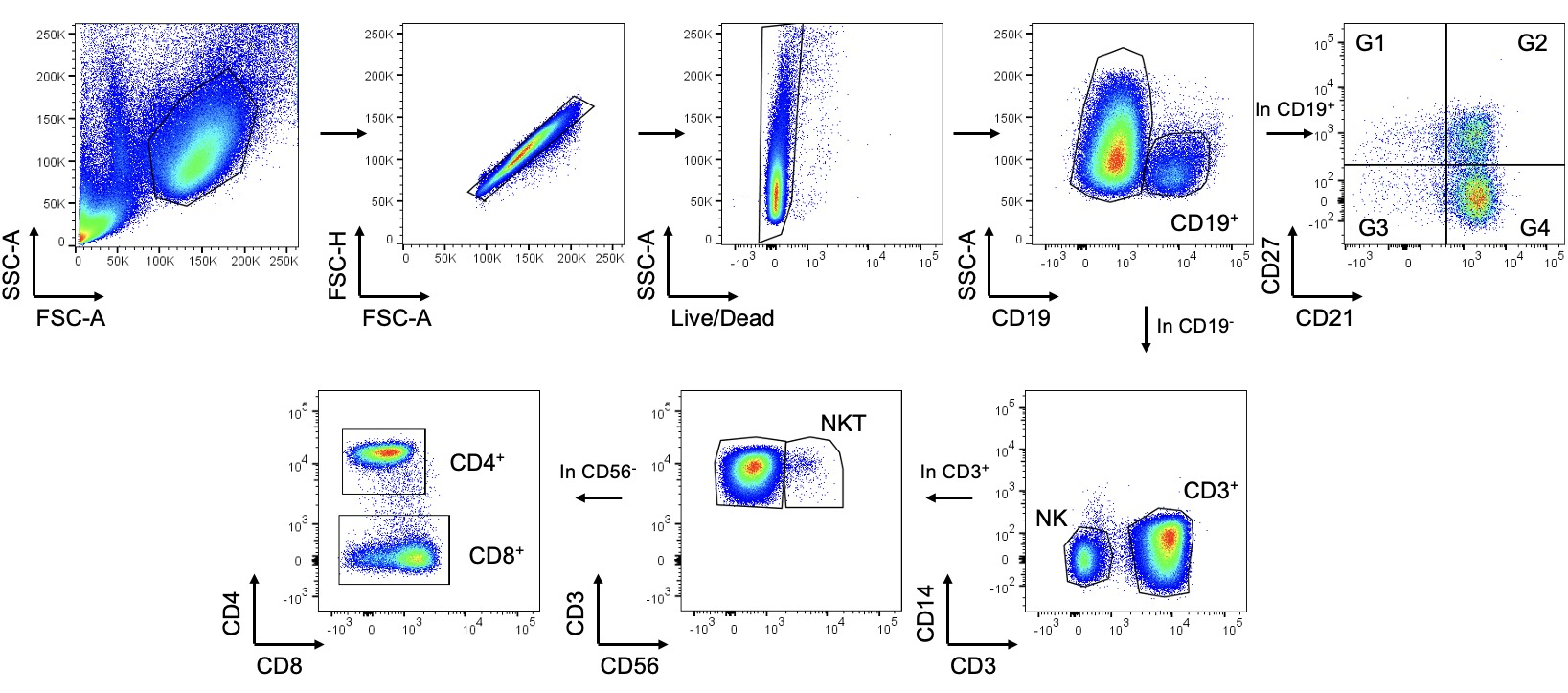

Supplement: S1 Fig — Cells were pre-gated for live/dead and then identified as follows: CD19+ B cells, activated memory B cells CD19+CD27+CD21- (G1), Classical memory B cells CD19+CD27+CD21+ (G2), Naïve B cells CD19+CD27-CD21+ (G4), Atypical memory B cells CD19+CD27-CD21- (G4), Live CD3+, NK cells (CD19-CD3-CD14-), NKT cells (CD19-CD3+CD56+), CD4+ and CD8+ T cells (CD3+CD56-). (TIF) [file pntd.0011229.s001.tif]
